# New investigation of anti-inflammatory activity of *Polycladia crinita* and biosynthesized selenium nanoparticles: isolation and characterization

**DOI:** 10.1186/s12934-023-02168-1

**Published:** 2023-09-05

**Authors:** Alanood S. Almurshedi, Thanaa A. El-Masry, Hend Selim, Mostafa M. El-Sheekh, Mofida E. M. Makhlof, Basmah N. Aldosari, Iman M. Alfagih, Bushra T. AlQuadeib, Salma S. Almarshidy, Maisra M. El-Bouseary

**Affiliations:** 1 Department of Pharmaceutics, College of Pharmacy, King Saud University, Riyadh, Saudi Arabia; 2 Department of Pharmacology and Toxicology, Faculty of Pharmacy, Tanta University, Tanta, Egypt; 3 Department of Biochemistry, Faculty of Pharmacy, Tanta University, Tanta, Egypt; 4 Botany Department, Faculty of Science, Tanta University, Tanta, Egypt; 5 Botany and Microbiology Department, Faculty of Science, Damanhour University, Damanhour, Egypt; 6 Department of Pharmaceutical Microbiology, Faculty of Pharmacy, Tanta University, Tanta, Egypt

**Keywords:** *Polycladia crinita*, Macroalgae, Brown Algae, Selenium nanoparticles, Anti-inflammatory, Anti-oxidant, Carrageenan

## Abstract

**Background:**

Marine macroalgae have gained interest recently, mostly due to their bioactive components. *Polycladia crinita* is an example of marine macroalgae from the *Phaeophyceae* class, also known as brown algae. They are characterized by a variety of bioactive compounds with valuable medical applications. The prevalence of such naturally active marine resources has made macroalgae-mediated manufacturing of nanoparticles an appealing strategy. In the present study, we aimed to evaluate the antioxidant and anti-inflammatory features of an aqueous extract of *Polycladia crinita* and biosynthesized *P. crinita* selenium nanoparticles (PCSeNPs) via a carrageenan-induced rat paw edema model. The synthesized PCSeNPs were fully characterized by UV–visible spectroscopy, FTIR, XRD, and EDX analyses.

**Results:**

FTIR analysis of *Polycladia crinita* extract showed several sharp absorption peaks at 3435.2, 1423.5, and 876.4 cm^−1^ which represent O–H, C=O and C=C groups. Moreover, the most frequent functional groups identified in *P. crinita* aqueous extract that are responsible for producing SeNPs are the –NH2–, –C=O–, and –SH– groups. The EDX spectrum analysis revealed that the high percentages of Se and O, 1.09 ± 0.13 and 36.62 ± 0.60%, respectively, confirmed the formation of SeNPs. The percentages of inhibition of the edema in pretreated groups with doses of 25 and 50 mg/kg, i.p., of PCSeNPs were 62.78% and 77.24%, respectively. Furthermore, the pretreated groups with 25, 50 mg/kg of *P. crinita* extract displayed a substantial decrease in the MDA levels (P < 0.00, 26.9%, and 51.68% decrease, respectively), indicating potent antioxidant effect. Additionally, the pretreated groups with PCSeNPs significantly suppressed the MDA levels (P < 0.00, 54.77%, and 65.08% decreases, respectively). The results of immune-histochemical staining revealed moderate COX-2 and Il-1β expressions with scores 2 and 1 in rats pre-treated with 25 and 50 mg/kg of free extract, respectively. Additionally, the rats pre-treated with different doses of PCSeNPs demonstrated weak COX-2 and Il-1β expressions with score 1 (25 mg/kg) and negative expression with score 0 (50 mg/kg). Both antioxidant and anti-inflammatory effects were dose-dependent.

**Conclusions:**

These distinguishing features imply that this unique alga is a promising anti-inflammatory agent. Further studies are required to investigate its main active ingredients and possible side effects.

**Supplementary Information:**

The online version contains supplementary material available at 10.1186/s12934-023-02168-1.

## Background

Algae have numerous special qualities, including a quick rate of growth, a high biomass generation rate, and the ability to accumulate and reduce mineral ions. Both micro- and macroalgae can develop without the assistance of chemicals or fertilizers. Algae are harvested often throughout the year, unlike many other biological materials [1]. They can be used as a valuable nanofactory in a water medium at normal pressure, temperature, and pH [2]. *Polycladia crinita*, formerly referred to as *Cystoseira crinita*, is an example of marine macroalgae from the *Phaeophyceae* class, also known as brown algae [3]. Species belonging to this genus have been seen synthesise a variety of secondary metabolites, including polysaccharides, lipids, triacylglycerols, phlorotannins, phenolic substances, and terpenoids [4]. Various unique terpenoids including tetraprenyltoluquinol, triprenyltoluquinol, and tetraprenyltoluquinone derivatives were recovered from *C. crinita*. The former compounds showed potent antioxidant properties [4]. Fucosterol, the distinctive steroid of brown algae that was initially identified in *C. compressa*, was additionally detected in *C. crinita*. Fucosterol has been proven to have a number of biological activities, including radical scavenging, antimicrobial, antioxidant, anti-inflammatory, and anti-cancer properties [4]. Other biomolecules, such as fucoidans, were proven to be useful in preventing the formation of edema via strong radical scavenging ability [4]. As a result of the aforementioned, there is a wide range of naturally occurring molecules in *Cystoseira* that have a variety of biological potentials and could be exploited as scaffolding to form innovative pharmacological approaches.

Nanotechnology offers fresh, “intelligent,” health-protection strategies and, in fact, has a significant potential to enhance well-being [5]. The distinctive physicochemical characteristics of nanoparticles (NPs), which include their small size (1–100 nm), excellent stability, hydrophobic nature, and vast surface area, are primarily what have sparked this interest. The hydrophobic nature of NPs is crucial for optimum dispersion in water or serum and is also necessary to promote their interactions with cell membranes [6]. The nanominerals (such as Se and Zn) have a greater surface-area-to-volume ratio, offering a greater surface area for contact with cells [7]. Proteins attach to the surface of nanominerals when they are placed in biological media, giving them a distinct “biological identity” or “protein corona,” which can affect both the distribution of the NPs and their possible toxic effects [8].

The three main categories of NP synthesis techniques are physical, chemical, and biological (also known as the “green way” or “green synthesis”). The most widely used commercial techniques for generating nanoparticles for a variety of NP applications are chemical ones. In addition, a wealth of research points to a potential environmental risk associated with nanotechnology and NP toxicity. The chemical method of NP synthesis involves the use of noxious compounds that are harmful to both people and the surrounding ecosystem. The detrimental environmental effect of NP construction is reduced by using biological and environmentally conscious components [9]. Employing plants or plant components to bioreduce metal ions into their elemental form is a key component in the green production of nanoparticles [9]. The “green synthesis” is more affordable, effective, and can be quickly scaled up to carry out greater activities. Hence, it is preferred to synthesize nanoparticles using environmentally friendly processes.

The byproducts of algal metabolism are capable of producing metal or metal oxide nanoparticles by reduction, capping, and stabilization of metal initial molecules [10]. Brown macroalgae are notable for their robust biomass, which generates a variety of physiologically active chemicals that can be employed as efficient reducing and stabilizing agents during the production of nanoparticles. Regarding the great number of such bio-active marine materials, macroalgae-mediated manufacturing of nanoparticles is therefore a promising strategy [3]. Since then, González-Ballesteros and coauthors revealed that the marine macroalga *Polycladia baccata* was capable of biologically synthesizing gold nanoparticles [11], and Touliabah et al*.* showed that *Polycladia myrica* was capable of biologically manufacturing selenium nanoparticles [12]. According to research studies, *P. crinita* extract has antimicrobial, antioxidant, cytotoxic, antiviral, anti-inflammatory, and antiproliferative properties [4].

Selenium (Se) is a vital element for the well-being of both animals and humans, where it mediates the activity of multiple enzymes and halts free radical-induced tissue damage. Recently, there has been a vast growth in interest in nanoparticles and nanomaterials science for their novel applicability [13]. Selenium nanoparticles (SeNPs) are attracting more and more attention owing to their exceptional biological activity, chemical stability, and low toxicity. Besides, coating Se nanoparticles with biological substances such as natural extracts has enhanced the biological activities of the NPs [13–16].

Inflammation is a vital process that defines the human body’s defense response, which acts to remove/neutralize any harmful event like microbial infection, chemical irritants, and tissue injury [17, 18]. As a result, inflammation comes with typical signs including pain, redness, and swelling, which indicate the aggravation of various inflammatory mediators. One of the main inflammatory markers that participate in the process of inflammation is prostaglandin E2, which is produced by the cyclooxygenase-2 enzyme and drives multiple pathways that trigger inflammation. Another pro-inflammatory mediator is interleukin-1 beta (IL-1β), which is generated and triggers multiple cascades of inflammation [19]. Additionally, reactive oxygen species are considered critical contributors to inflammation, where they activate pro-inflammatory cytokines and interleukins production and arouse oxidative stress-mediated tissue damage [20, 21].

Carrageenan-induced paw edema is a frequently used model to evaluate the anti-inflammatory potential of numerous natural compounds [22]. Although there is wide use of nonsteroidal anti-inflammatory drugs (NSAIDs) in managing inflammation, they still have the disadvantage of causing untoward side effects, exemplified by renal, gastrointestinal, and cardiac toxicity, which correlates with long-time usage. Thus, finding a safe and effective anti-inflammatory agent has become a priority [23].

Exploiting the superior pharmacological features of the bioactive compounds extracted from the exceptional brown algae *P. crinita* as well as its unique ability to bioreduce metal ions to fabricate nanoparticles, in addition to the deficiency of research regarding biosynthesized selenium nanoparticles as an effective drug delivery system, were strong motivations to conduct this research study. The green synthesis of SeNPs by *P. crinita* has not yet been reported. We designed this study in order to extract the distinctive biomolecules from *P. crinita* and make use of these compounds to biosynthesize SeNPs. Our study design also involved the full characterization of *P. crinita* extract and selenium nanoparticles (SeNPs) loaded *P. crinita* extract. To the best of our knowledge, this manuscript is the first to investigate the anti-inflammatory effect of *P. crinita* algae, either free or loaded on SeNPs, in carrageenan-induced rat paw edema, besides elucidating possible underlying mediators that are involved in such an effect.

## Materials and methods

### Materials

All the used chemicals were purchased from Sigma Aldrich in Cairo, Egypt, and were of analytical quality. Sodium selenate (Na_2_SeO_4_) was used as a precursor for SeNPs synthesis. Carrageenan was obtained from Merck (Kenilworth, NJ, USA).

### Macroalgae biomass collection

Biomass of *Polycladia crinita* was collected from the Gulf of Suez, Egypt coast. The alga was identified by Dr. Fekry Mourad, a researcher at NIOF Egypt. The identification of collected samples was performed according to the methods of Aleem [24–26] and was confirmed using the Algae Base website [27].

### Macroalgae aqueous extracts preparation and biosynthesis of SeNPs

*Polycladia crinita* biomass was collected, rinsed with distilled water to get rid of any associated detritus and remains, and then washed numerous times with water to get rid of the sea salts. The epiphytes were manually taken away. Then, the samples were dried in the shade at room temperature, followed by oven drying at 60 °C for 15 min, and then sample grinding was done into fine particles using an electric mixer. *P. crinita* powder weighing approximately ten grams was added to 100 ml of H2O, thoroughly agitated, and heated using a magnetic stirrer for 120 min at 60 °C. The mixture was then centrifuged for 10 min at 1500 rpm [28], and the supernatant has been utilized as a reducing and stabilizing agent for SeNPs by adding 1 mM of Na_2_SeO_4_ to the algal extract in a ratio of 1:9. After 24 h of incubation in dark, shaking conditions, the color of the mixture changed from pale brown to dark brown, indicating the synthesis of PCSeNPs [29]. The resultant PCSeNPs were then centrifuged at 10,000 rpm for 30 min, washed with distilled water, treated using pure ethanol, heated at 50 °C, maintained in a sealed flask, and then employed for any subsequent studies [12].

### Characterization of the synthesized PCSeNPs

The maximal surface plasmon resonance (SPR) of biosynthesized PCSeNPs was determined by measuring the absorbance spectra with a UV–Vis spectrophotometer (Thermo Scientific Evolution TM 300, Thermo Fisher Scientific, Waltham, MA, USA) in the 200–800 nm range [30]. Fourier transform infrared (FTIR) spectroscopy (PerkinElmer System 2000 instrument FT-IR model) was used to investigate the contribution of functional groups observed in the aqueous extract of *P. crinita* in the reduction and stabilizing processes. The PCSeNPs sample was combined with KBr and pressed in order to create a disc that could be scanned between 400 and 4000 cm^−1^ [31].

Transmission electron microscopy (TEM; JEM 2100, JEOL Ltd., Tokyo, Japan) was employed to examine the shapes and sizes of biosynthesized PCSeNPs. The TEM grid was treated with a drop of MgO-NPs colloidal solution, and any surplus solution was removed by blotting the grid against paper. Before being mounted on the TEM holder, the loaded grid was vacuum desiccated for 24 h [32]. Scanning Electron Microscopy coupled with energy-dispersive X-ray (SEM–EDX) (JSM 6490 LV, JEOL, Ltd., Tokyo, Japan) was employed for examining the elemental composition of the PCSeNPs sample [33].

The XRD 6000 detector (Shimadzu Corp., Kyoto, Japan) was utilized to examine the X-ray diffraction (XRD) data at a 2θ degree of 0°–80°. The x-ray source was a 2.2 KW Cu anode, and the operating parameters were voltage at 30 kV, current at 10 mA, and k = 1.54184. The Debye–Scherrer equation was employed to calculate the particle size [34] based on XRD analysis. (D = λk/βcosθ), where k was Scherrer’s constant, roughly equaling 0.9, the X-ray wavelength (λ), 1.54060 nm, the half-maximum intensity (β), and the Bragg diffraction angle, which was calculated using the formula = (θ1 − θ2)π/180. The line width of the (113) reflection in XRD was specifically employed to confirm the material's particle size. Finally, the measurements of the size, polydispersity index (PDI), and zeta potential of the samples were carried out by Zeta Plus (Brookhaven, USA) at 25 °C. Triplicate analyses of each sample were performed.

### Experimental model for inflammation induction

Inflammation was induced by injecting the subplanter’s right hind paw with 0.2 ml of subcutaneous (SC) injection of freshly prepared 1% carrageenan solution. To preserve control, the left hind paw was not injected [35].

#### Animals

Fifty-four male Wistar albino rats were obtained from the animal house located at the faculty of veterinary medicine, Cairo University, Egypt. The rats were supplied with filtered water and a standard pelleted diet (IBEX feed for research animals, Ibex International Co., Ltd., Cairo, Egypt) at 25 ± 2 °C and 12-h light/dark cycles for a 2-week acclimatization period. Healthy male rats of approximately the same weight (170–200 g) were chosen for the experiment. The in vivo methods and protocol were endorsed by the Research Ethical Committee of the Faculty of Pharmacy, Tanta University, Egypt, as aligned with the standard regulations for handling and caring for laboratory animals (TP/RE/11/22p-0065).

#### Experimental design and animal groups

Rats were randomly assigned into nine groups (6 rats per group) (see Additional file 1: Fig. S1). The animal care providers, the lab technicians, and the histopathology experts were blinded regarding which rats received treatment and the nature of the treatment provided to the animals to avoid any bias. Group I is the negative control group, while Group II is the positive control group. In both groups (I and II), the rats were injected i.p. with 0.9% saline (10 ml/kg). In the cases of Groups III and IV, the rats were pretreated with *P. crinita* extract that was injected (i.p.) in doses of 25 and 50 mg/kg, respectively. Concerning Groups V and VI, the rats were pretreated by injection (i.p.) of selenium nanoparticles (SeNPs) loaded *P. crinita* extract in doses of 25 and 50 mg/kg, respectively. While the rats in Groups VII and VIII were injected i.p. with *P. crinita* extract (50 mg/kg) and PCSeNPs (50 mg/kg), respectively, the last group (Group IX) involved the rats that were pretreated with SeNPs placebo. The amount of extract loaded on SeNPs in groups V and VI was equivalent to the free *P. crinita* given to groups III and IV. The doses of *P. crinita* extract were chosen based on the doses mentioned in earlier studies, which were confirmed with a pilot study [36]. After 1-h, acute inflammation was induced by carrageenan injection in rats of all groups except groups I, VII, and VIII. After 6 h, animals were anesthetized with isoflurane, and the euthanasia of rats was performed by cervical dislocation (CD) according to the American Veterinary Medical Association (AVMA) Guidelines for the Euthanasia of Animals (2020 Edition). Both left and right paws were cut at the same point and weighed [37]. The average edema weight was determined using the difference between the right and left paw weights [38].

#### Sample collection

The paws were weighted, and after that, the right paw tissue was taken and divided into two groups. One group was kept in a − 80 freezer for the measurement of malondialdehyde (MDA) and prostaglandin E2 (PGE2). The other one was kept in formalin, which was used for further histopathological and immunohistochemical staining and examination.

##### Measurement of malondialdehyde (MDA)

Tissue MDA levels were measured using “Biodiagnostic kits” (Giza, Egypt) at 540 nm, and the steps were conducted as per the manufacturer’s instructions.

##### Measurement of prostaglandin E2 (PGE2)

To determine PGE2 protein levels, an enzyme-linked immunosorbent assay (ELISA) kit was obtained from Bio-Neovan Co., Ltd. (Beijing, China). The absorbance was measured at 450 nm using an ELISA reader (Sunrise, Switzerland).

#### Histological and immuno-histochemical examination

The paw tissue was taken out of formalin, located in paraffin wax, sectioned (with a 5-µm thickness), and stained with hematoxylin and eosin. Sections were then examined by a light microscope.

For immune-histochemical staining, the paw COX-2 and Il-1β expressions were investigated by immunostaining the tissues using “AB-clonal Technology kits” (Woburn, MA, USA). Based on the percentages of positive staining, the results were scored. These scores are as follows: 0 denotes the absence of cells that stained positively, and 1 denotes the presence of cells that stained positively in a range of 25% to 75%. Score 2 denotes the presence of cells that are 11–50% immunostained, Score 3 the presence of cells that are 51–75% immunostained positively, and Score 4 the presence of cells that are 76–100% immunostained positively [39].

### Statistical analysis

The results were represented graphically as the mean standard deviation (SD). Following a post hoc test, a one-way analysis of variance (ANOVA) was applied to compare various groups. In the event where *p* < 0.05, the data was regarded as significant.

## Results

### FTIR analysis of *Polycladia crinita* extract

**Fig. 1:**
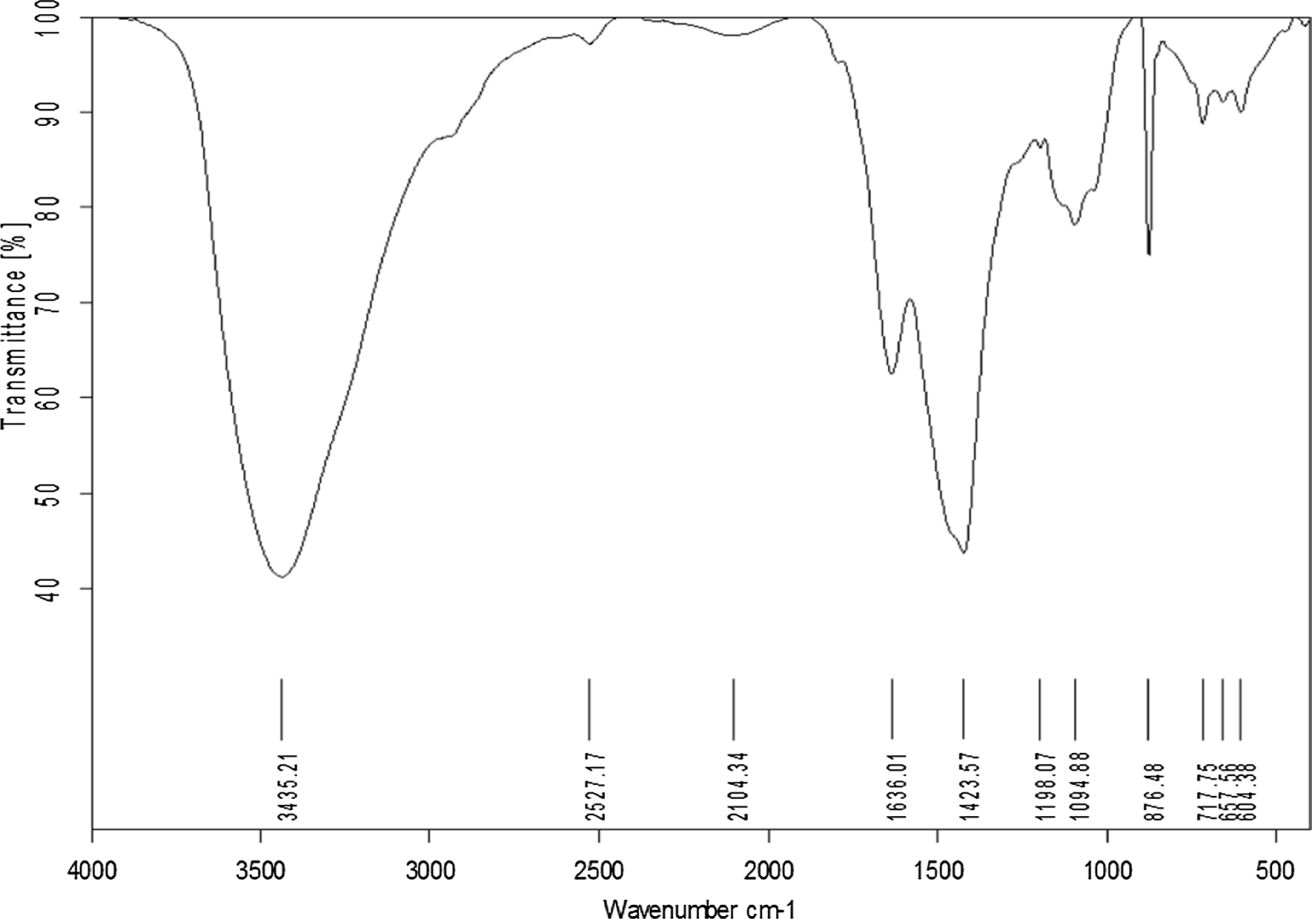
FTIR spectra of *Polycladia crinita* aqueous extract

The main sharp absorption peaks were at 3435.2, 1423.5, and 876.4 cm^−1^ which represent O–H, C=O and C=C groups; also, there were a lot of weak and broad bands, such as the bands at 2527.16, 1636, 1198, and 604.3 that are accountable for the resonance of the –NCS, C=O, N–H, and C=S groups (Fig. 1).

### *Polycladia crinita* selenium nanoparticles (PCSeNPs) characterization

At the beginning of the synthesis reaction, 1 mM of sodium selenate solution was added to synthesize SeNPs in an Erlenmeyer flask in a ratio of 1:10. A control solution of 1 mM sodium selenate solution without marine algae extract was maintained at the same concentration. At the beginning of the experiment, the solution had a light brown color and did not exhibit any color change. The darkening of the mixture’s color after 48 h of reaction served as visible confirmation that sodium selenite had been reduced.

#### UV–Vis spectroscopy analysis

**Fig. 2:**
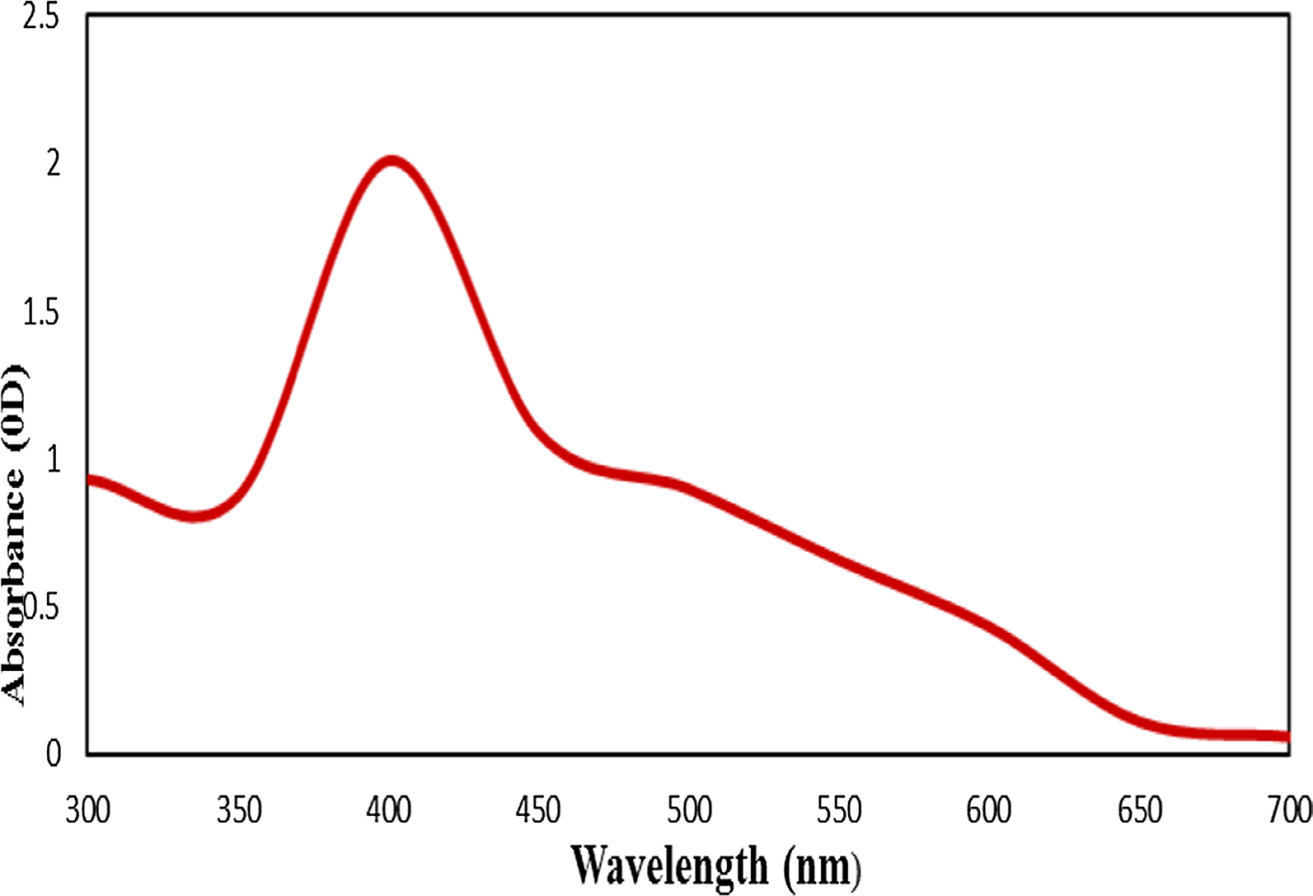
The UV–Visible spectrum of *Polycladia crinita* mediated selenium nanoparticles (PCSeNPs)

The synthesis of SeNPs using *P. crinita* extract was confirmed by the characterization of selenium nanoparticles based on surface plasmon resonance (SPR) vibrations observed at 400 nm. As shown in Fig. 2, the absorbance of the selenium SPR band, which ranged from 350 to 450 nm, steadily increased over a period of 48 h. It was clear from the broadening of the peak that the particles were polydispersed. The peak intensity also increased until the maximum absorbance was reached at 400 nm.

#### FTIR of *Polycladia crinita* selenium nanoparticles (PCSeNPs)

**Fig. 3:**
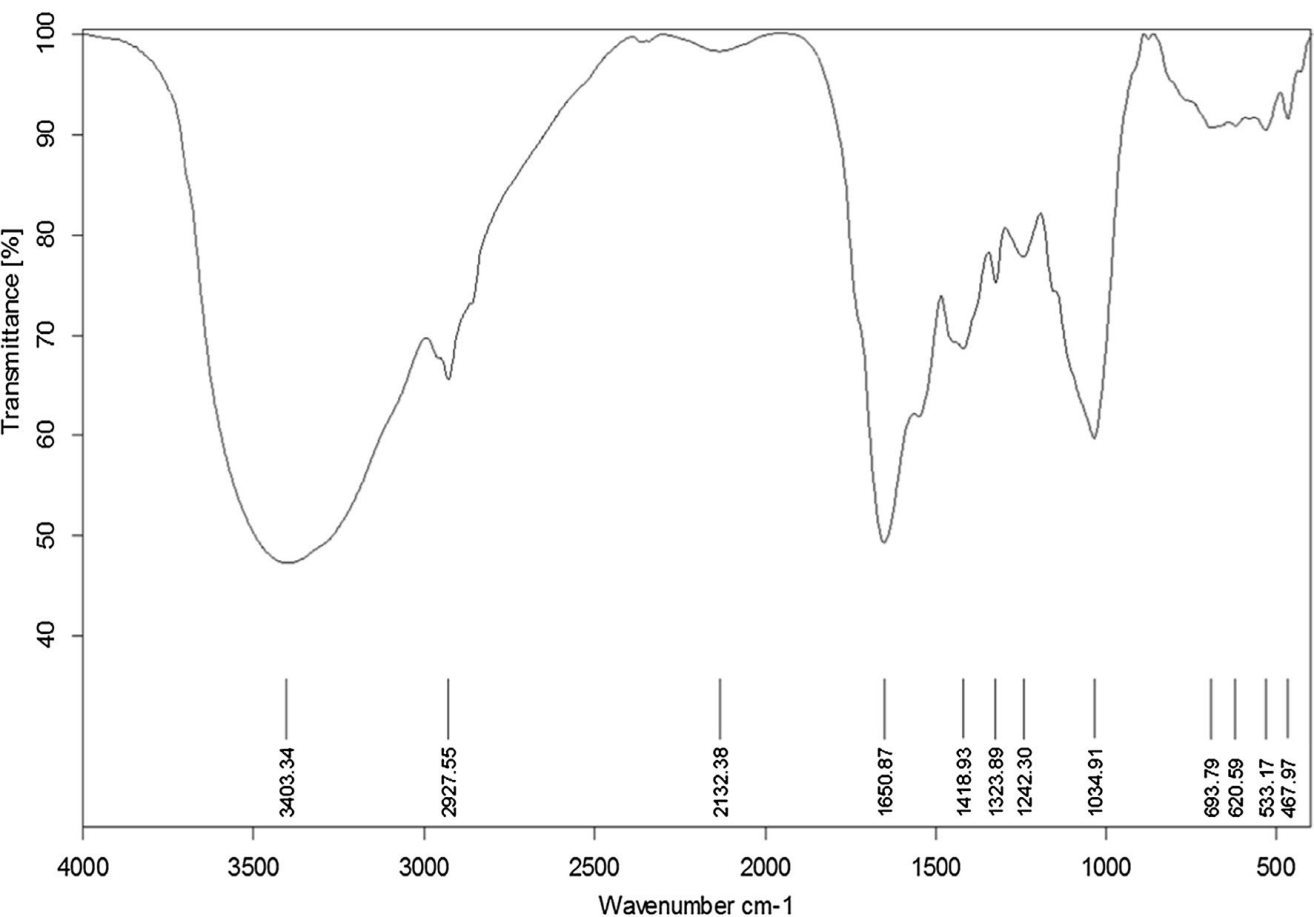
FTIR spectra of *Polycladia crinita* mediated selenium nanoparticles (PCSeNPs)

Figure 3 illustrates the main absorption bands of the formed selenium nanoparticles. There is a large degree of similarity between *P. crinita* and PCSeNPs FTIR figures as nearly the same sharp absorption bands occur, with minor degree of shifting, at 3403.3, 1650.8, and 620.5, with the presence of some peaks from 620.5 to 467.9666 that indicate the resonance of SeNPs.

#### Transmission electron microscopy (TEM)

**Fig. 4:**
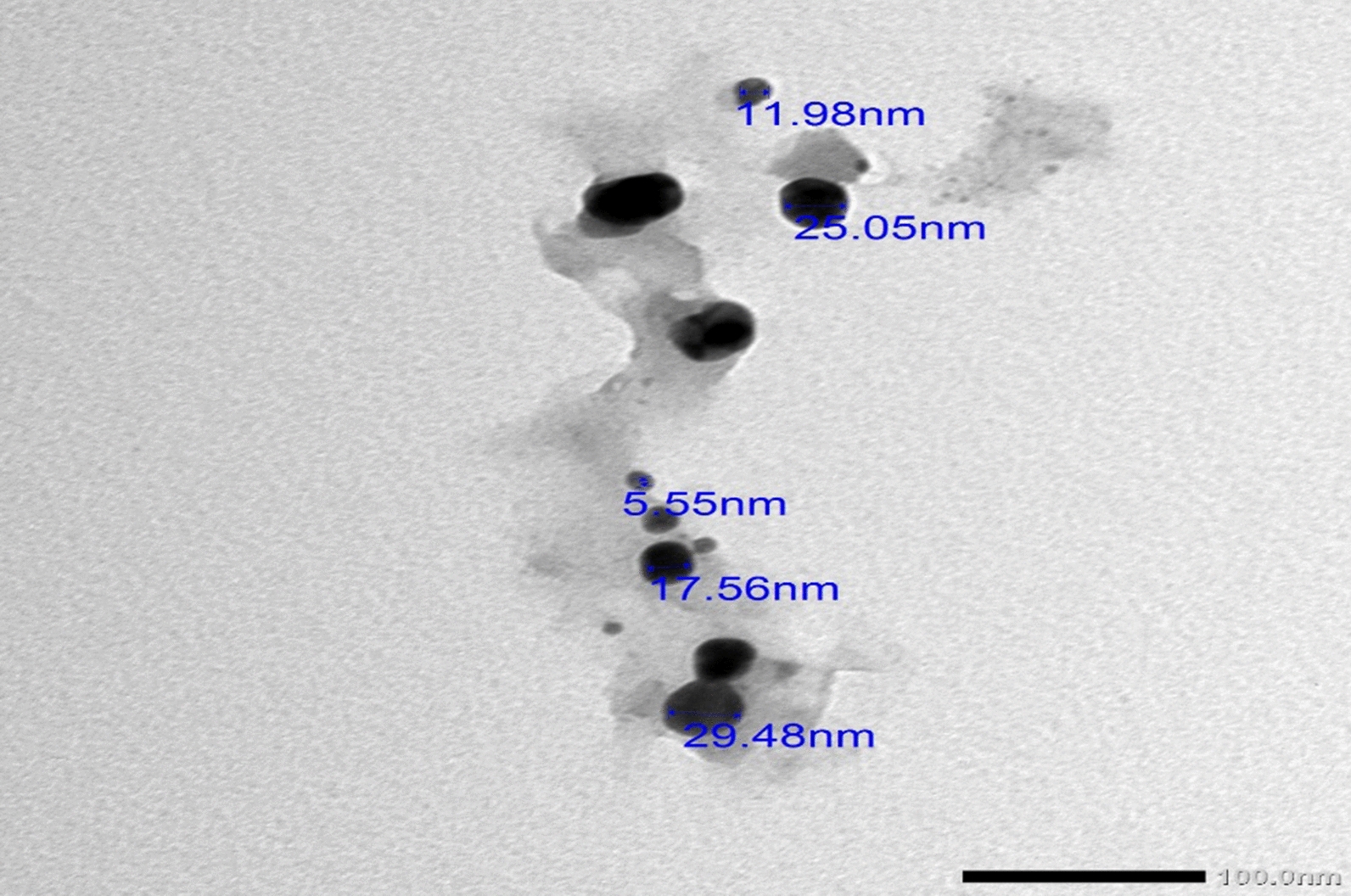
TEM image for *Polycladia crinita* mediated selenium nanoparticles (PCSeNPs)

The TEM image showed the smooth, sphere-shaped selenium nanoparticles synthesized by the reducing power of *P. crinita* extract for sodium selenite (Fig. 4). The selenium nanoparticles have an average size of 5.55–29.48 nm and a crowded background.

#### Scanning electron microscopy (SEM)

**Fig. 5:**
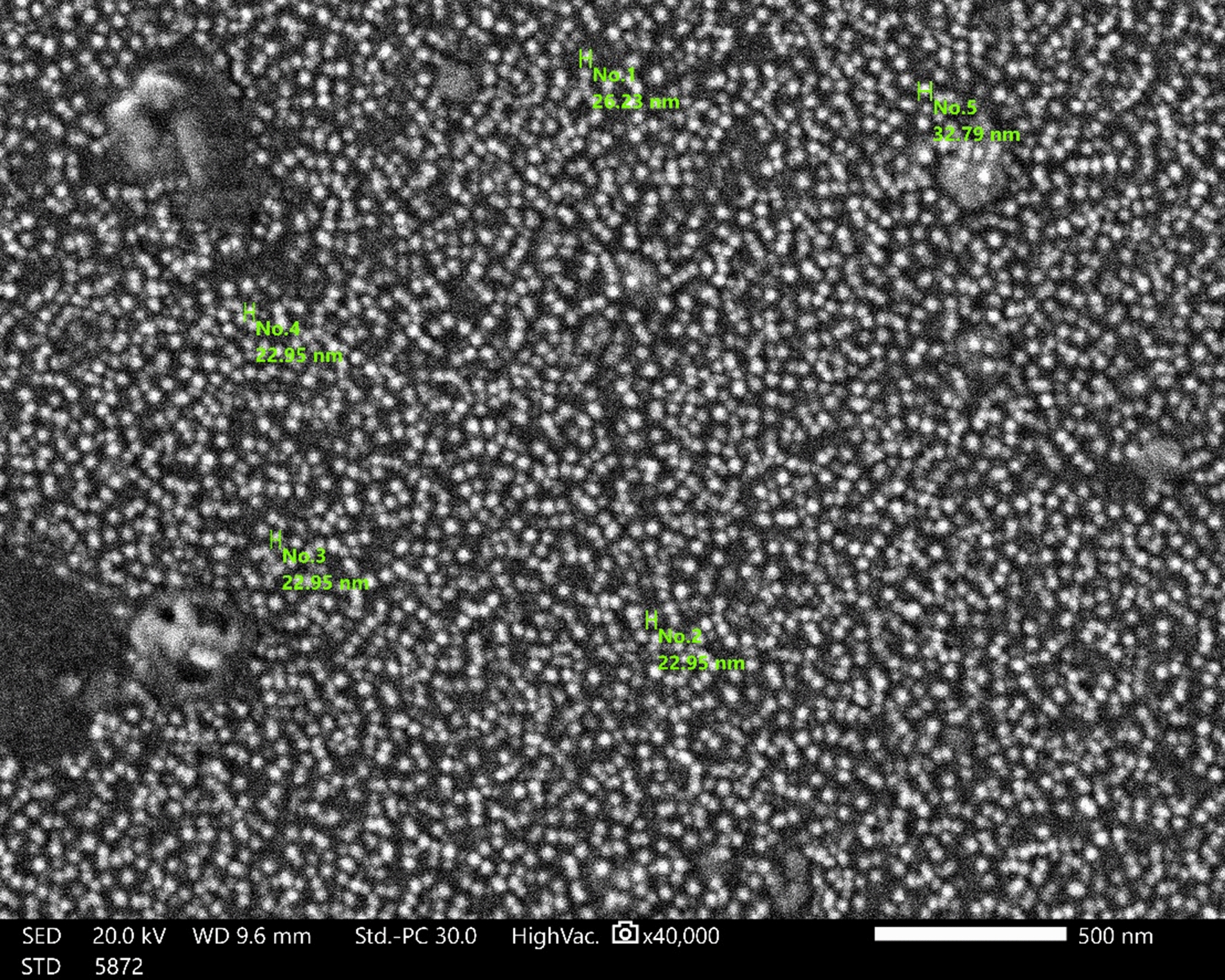
SEM image for *Polycladia crinita* mediated selenium nanoparticles (PCSeNPs)

The high-density selenium nanoparticles produced through processing *P. crinita* extract were detectable in the SEM image, which further supported the development of selenium nanostructures (Fig. 5). The average mean size of the selenium nanoparticles appears to range between 21.05 and 22.95 nm.

#### energy-dispersive X-ray analysis

**Fig. 6:**
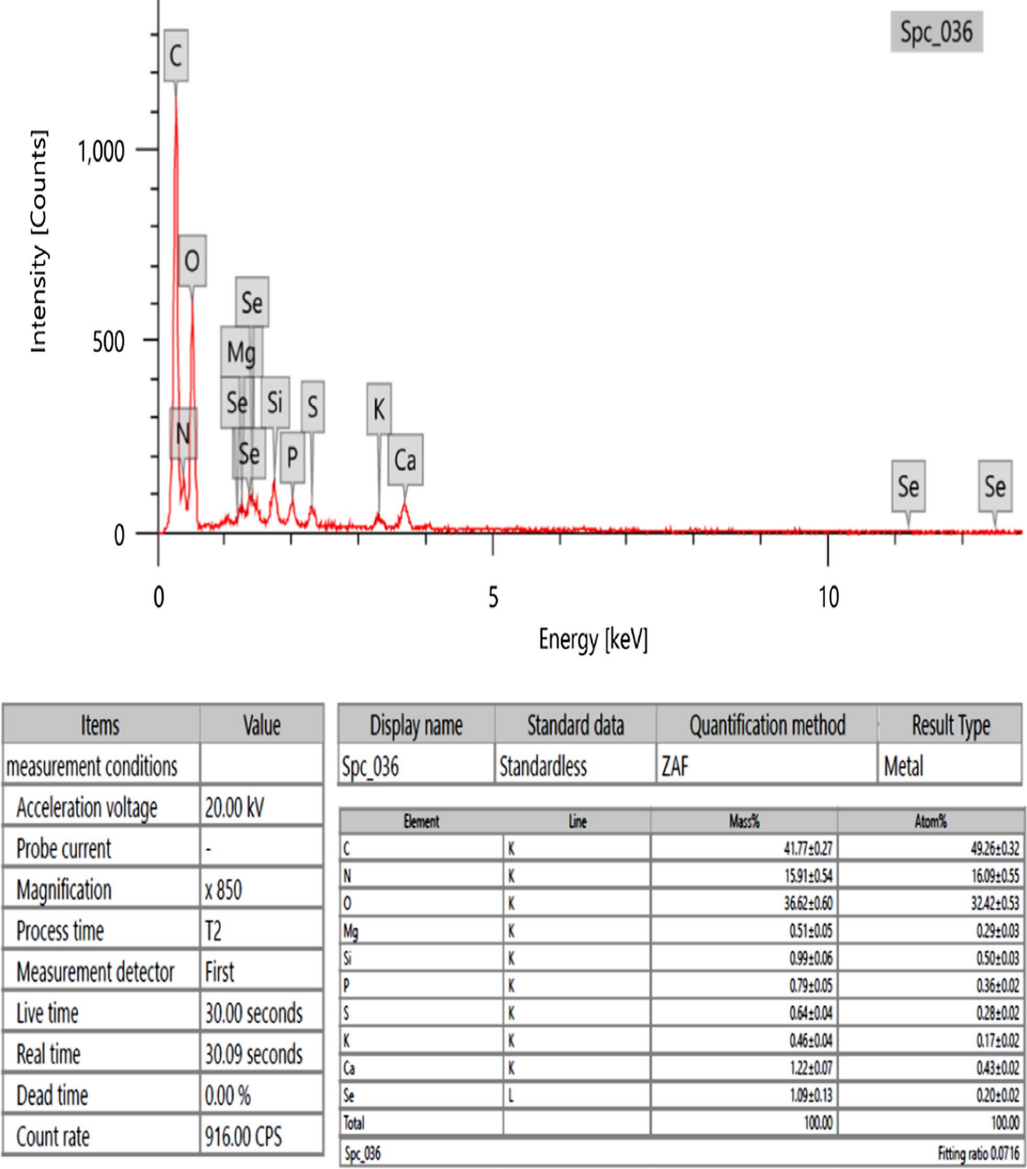
EDX analysis for *Polycladia crinita* mediated selenium nanoparticles (PCSeNPs)

Energy-dispersive X-ray spectroscopy was used to identify the components and atomic compositions of SeNPs manufactured by aquatic algae (Fig. 6). The EDX spectrum analysis revealed that the high percentages of Se and O, 1.09 ± 0.13 and 36.62 ± 0.60%, respectively, confirmed the formation of selenium nanoparticles with the presence of many other elements, like Mg, S, K, P, and C, that came from the algal extract components.

#### X-ray diffraction (XRD) analysis

**Fig. 7:**
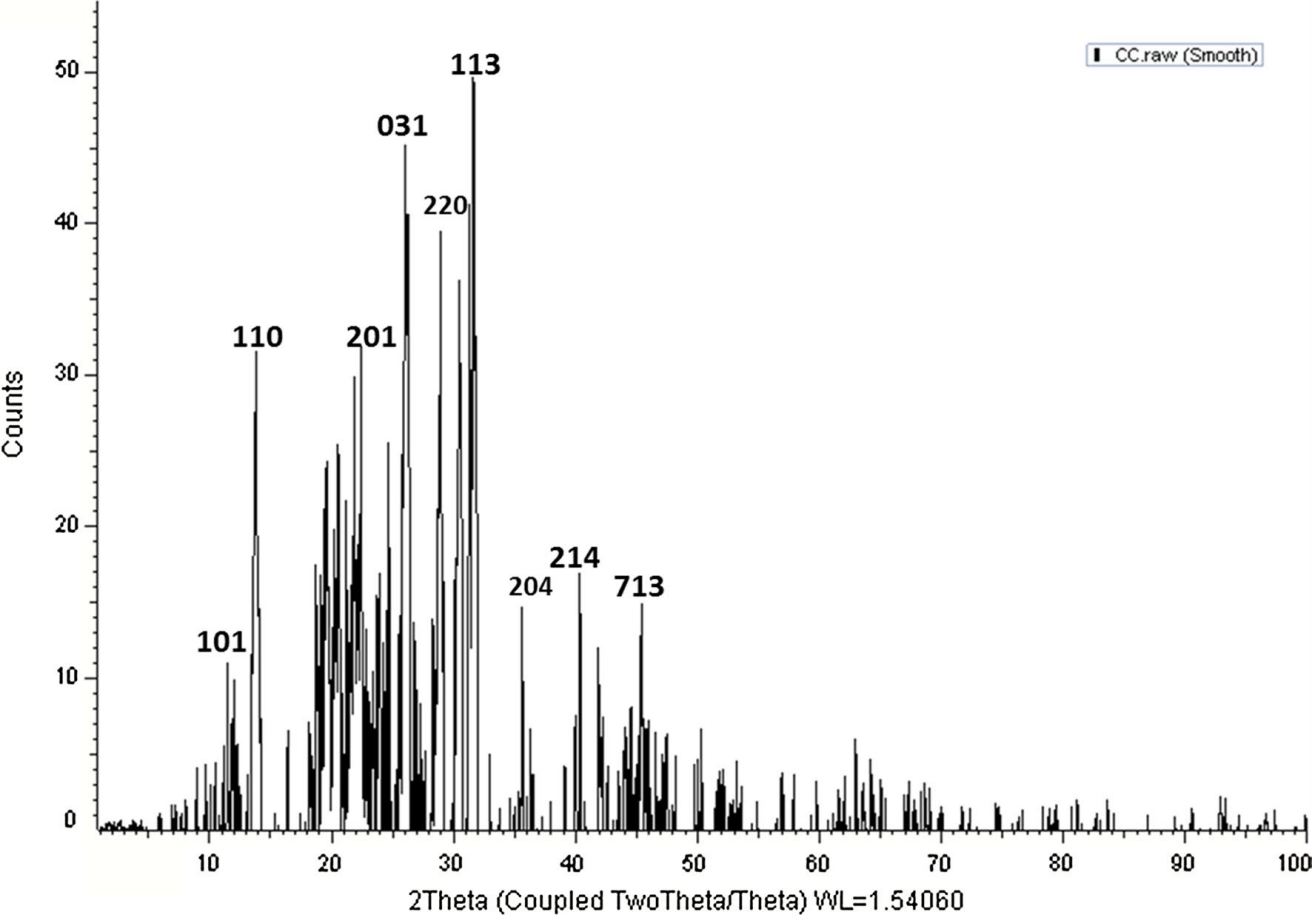
XRD spectra of *Polycladia crinita* mediated selenium nanoparticles (PCSeNPs)

The XRD pattern depicted in Fig. 7 was employed to study the crystallinity of physio-synthesized SeNPs. There are nine intense peaks at 2θ values of 11.5°, 14.2°, 22.43°, 26.33°, 28.64°, 32.13°, 36.41°, 40.2° and 45.92° which corresponded to (101), (110), (201), (031), (220), (113), (204), (214) and (713), respectively.

#### Zeta potential

**Fig. 8:**
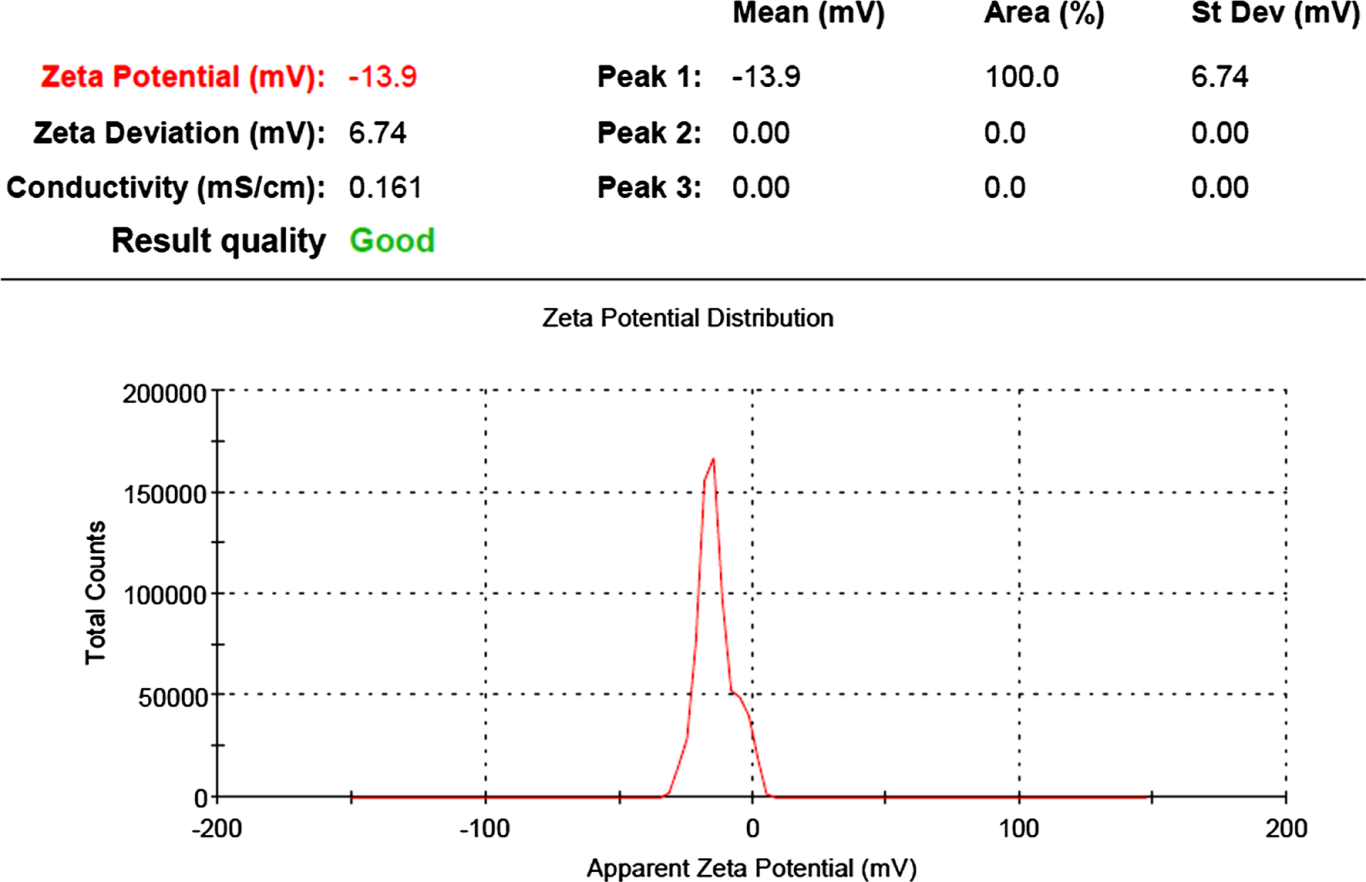
Zeta potential of *Polycladia crinita* mediated selenium nanoparticles (PCSeNPs)

The zeta potential (ζ) does not represent the real charge on each molecule, but rather an estimation of the electric double layer generated in the solution by the surrounding ions. It was confirmed that the formulated PCSeNPs had a negative charge, which meant that the SeNPs showed greater stability without aggregation. In the current study, the zeta potential was charged at − 13.9 mV, the peak contained 100.0% of the area, and the zeta deviation was 6.74 mV with 0.161 mS/cm conductivity (Fig. 8).

### Hand paws edema weight changes

**Fig. 9:**
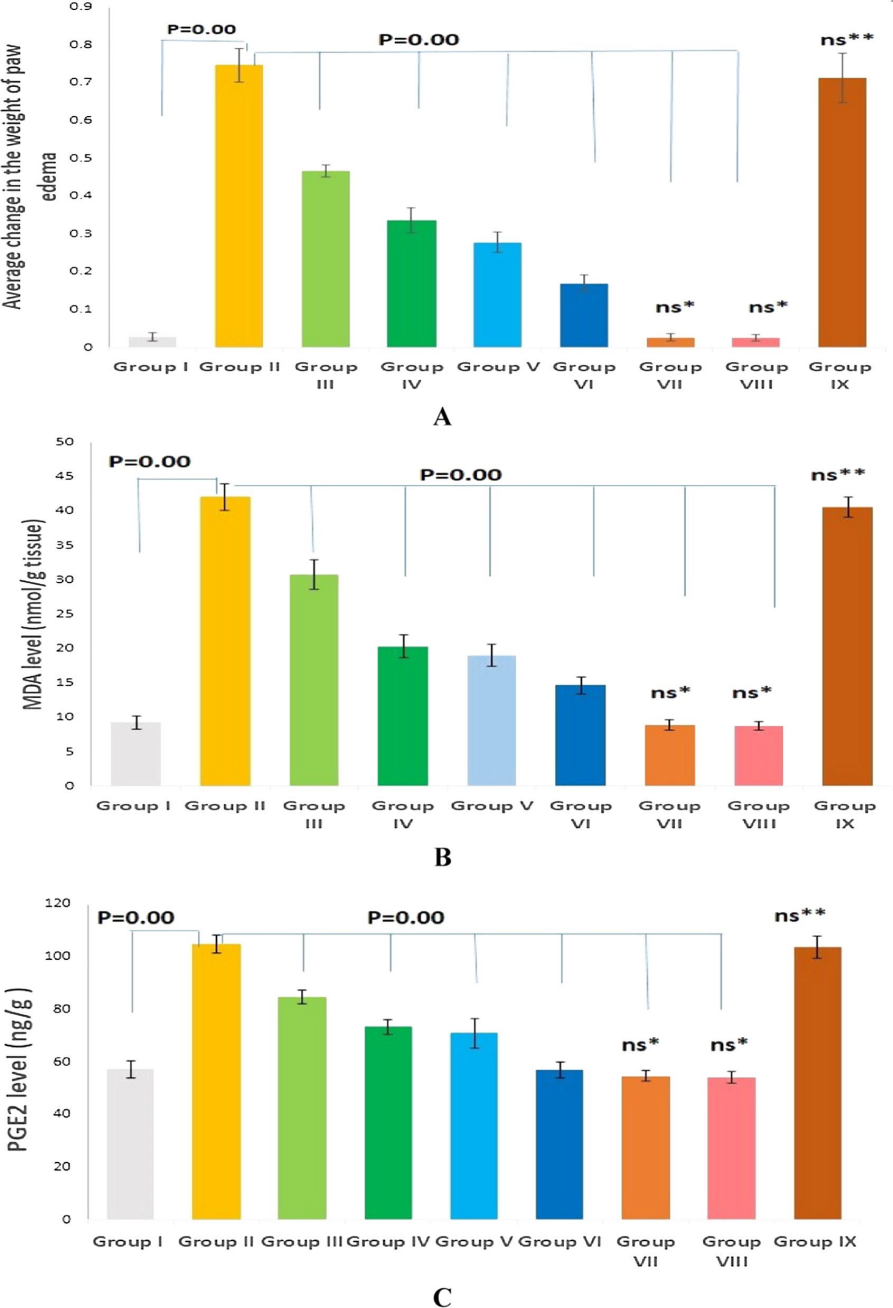
**A** Effect of various treatments on average paw edema weight changes. **B** Effect of various treatments on MDA levels. **C** Effect of various treatments on PGE2 levels. Data are expressed as mean ± standard deviation (SD), n = 6/group. ns* and ns** indicate not significant when compared to the negative and positive controls, respectively

As shown in Fig. 9A, the average paw edema weight was remarkably increased (P < 0.00) in Group 2 (the positive control), compared to the normal control. On the other hand, groups III, IV, V, and VI showed a significant reduction in the average paw edema weight (P < 0.00; 37.34%, 55.02%, 62.78%, and 77.24% decreases, respectively), in comparison with group II. Groups VII and VIII revealed no significant change in the average paw edema weight (P > 0.05) when compared with group I. Similarly, Group IX (SeNPs placebo) exhibited no marked change relative to Group II.

### Effect of treatments on paw tissue MDA levels

As speculated in Fig. 9B, group II exhibited a substantial increase in the tissue level of MDA (4.53-fold increase, P < 0.00). However, the pretreated groups (III and IV) exhibited a significant decrease in the MDA levels (P < 0.00, 26.9%, and 51.68% decrease, respectively). Likely, the groups pretreated with SeNPs loaded with the extract significantly suppressed the MDA levels (P < 0.00, 54.77%, 65.08% decreases, respectively), relative to the positive control group. There is no significant difference between group IX and the positive control.

### Effect of pre-treatments on paw PGE2 level

Investigations were performed on how different pre-treatments affected the tissue content of PGE2. The positive control group exhibited markedly increased PGE2 levels, relative to the normal control (Fig. 9C). On the contrary, groups pretreated with the free extract (Groups III and IV) revealed a significant reduction (P < 0.00) in the PGE2 content with a percentage of 19.27% and 30.2%, respectively. In comparison with the positive control, Groups V and VI (where rats were pre-treated with the extract loaded on SeNPs) showed a substantial decrease in the PGE2 levels, with percentages of 32.38% and 45.75%, respectively.

### Histopathological evaluation

**Fig. 10:**
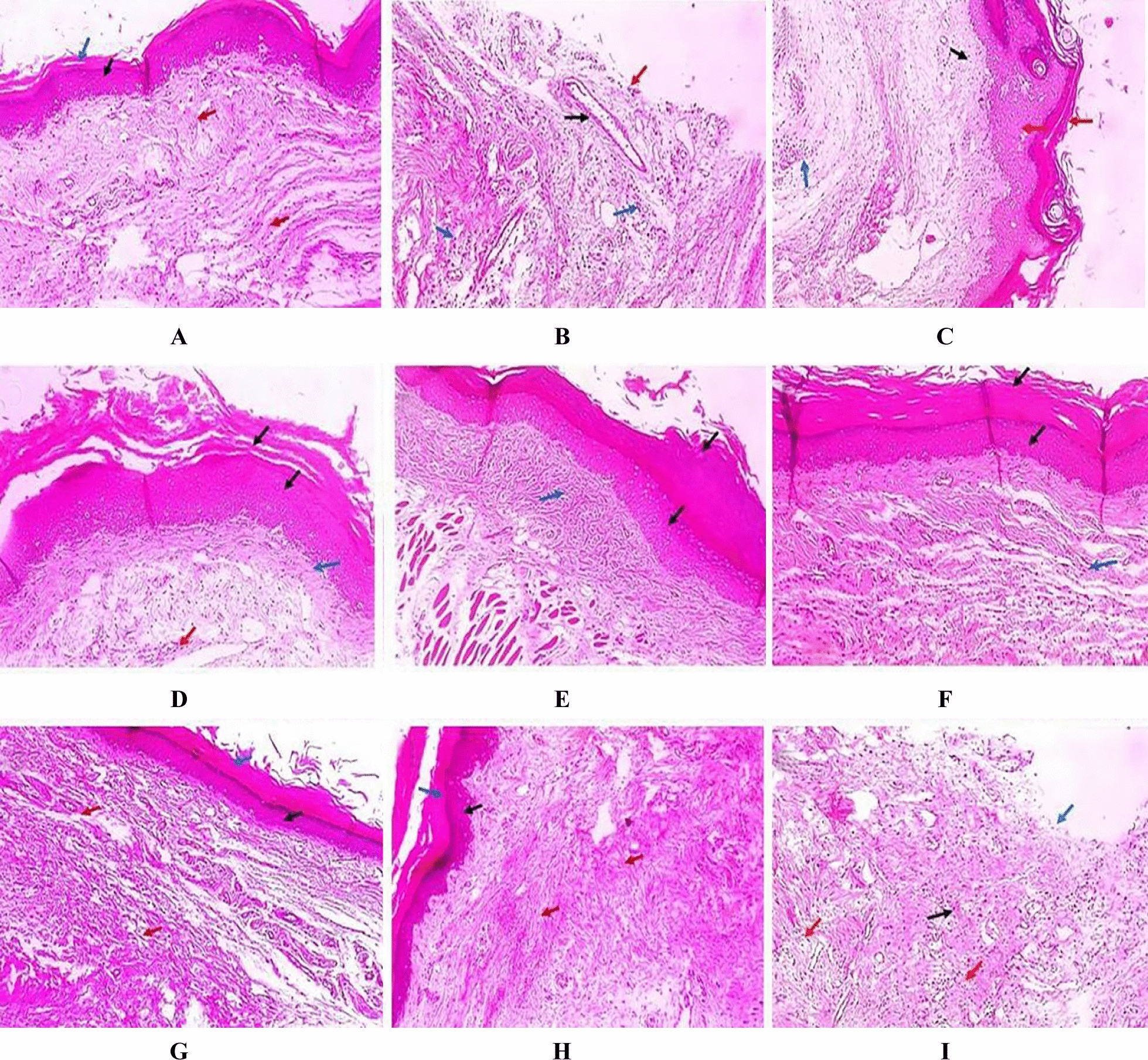
Hematoxylin and eosin (H&E)-stained paw sections [×100]. **A** Group I (negative control) showed normal skin with epidermis of average thickness (black arrow), thick keratin (blue arrow) and underlying normal dermis (red arrows). **B** Group II (positive control) showed surface ulceration (red arrow) with underlying granulation tissue containing congested blood vessels (black arrow) with infiltration by inflammatory cells (blue arrows). **C** Group III (rats pre-treated with free extract 25 mg) showed mild dermal inflammation (blue arrow), the epidermis was mild thickened lined with mild keratosis (red arrows) with no collagenosis (black arrow). **D** Group IV (rats pre-treated with free extract 50 mg) showed few dermal inflammation (red arrow), the epidermis was thickened lined with excessive keratosis (black arrows) with mild collagenosis (blue arrow). **E** Group V (rats pre-treated with Se NPs loaded with extract 25 mg) showed no dermal inflammation; the epidermis was thickened lined with marked keratosis (red arrows) and moderate collagenosis (blue arrow). **F** Group VI (rats pre-treated with Se NPs loaded with extract 50 mg) showed no inflammation; the epidermis is thickened with excessive keratosis (black arrows) and excessive collagenosis (blue arrow). **G** Group VII (rats received free extract 50mg only) showed normal skin with epidermis of average thickness (black arrow), thick keratin (blue arrow) and normal dermis (red arrows). **H** Group VIII (rats received placebo SeNPs only) showed normal skin with epidermis of average thickness (black arrow), thick keratin (blue arrow) and normal dermis (red arrows). **I** Group IX (rats pre-treated with placebo SeNPs) showed surface ulceration (blue arrow) with underlying granulation tissue (black arrows) and infiltration of inflammatory cells (red arrows)

Hematoxylin and eosin (H&E) were employed to stain the skin sections of the different groups, and the results are shown in Fig. 10.

### Immuno-histochemical examination

**Fig. 11:**
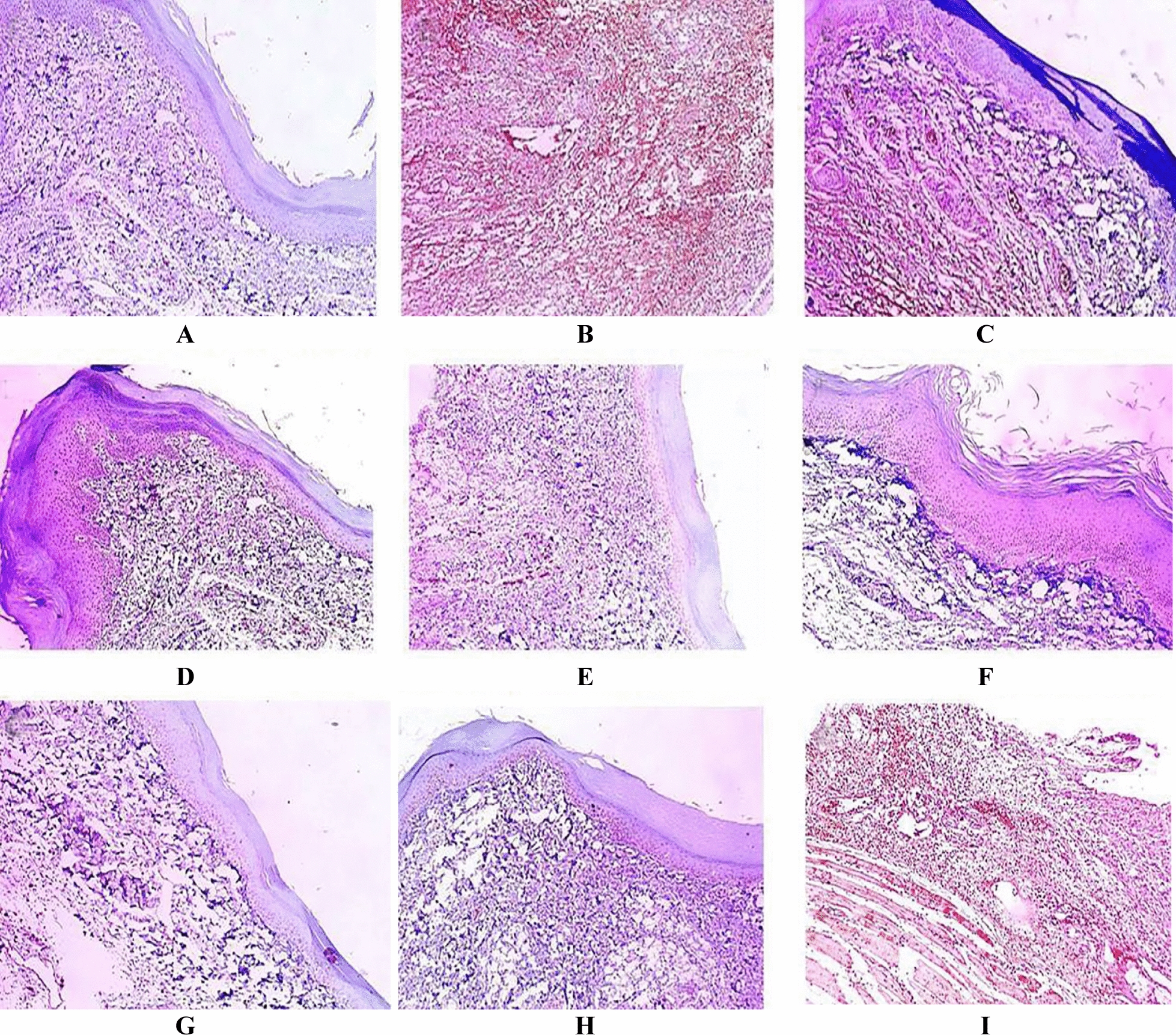
COX2 immuno-stained paw skin sections [×100]. **A** Group I (negative control) showed negative COX-2 expression with score 0. **B** Group II (positive control) showed marked COX-2 expression with score 4. **C** Group III (rats pre-treated with free extract 25 mg) showed moderate COX-2 expression with score 2. **D** Group IV (rats pre-treated with free extract 50 mg) showed moderate COX-2 expression with score 1. **E** Group V (rats pre-treated with SeNPs loaded with extract 25 mg) showed weak COX-2 expression with score 1. **F** Group VI (rats pre-treated with SeNPs loaded with extract 50 mg) showed negative expression with score 0. **G** Group VII Group (rats received free extract 50 mg only) showed negative COX-2 expression with score 0. **H** Group VIII (rats received placebo SeNPs only) showed negative COX-2 expression with score 0. **I** Group IX (rats pre-treated with placebo SeNPs) showed strong COX-2 expression with score 3

**Fig. 12:**
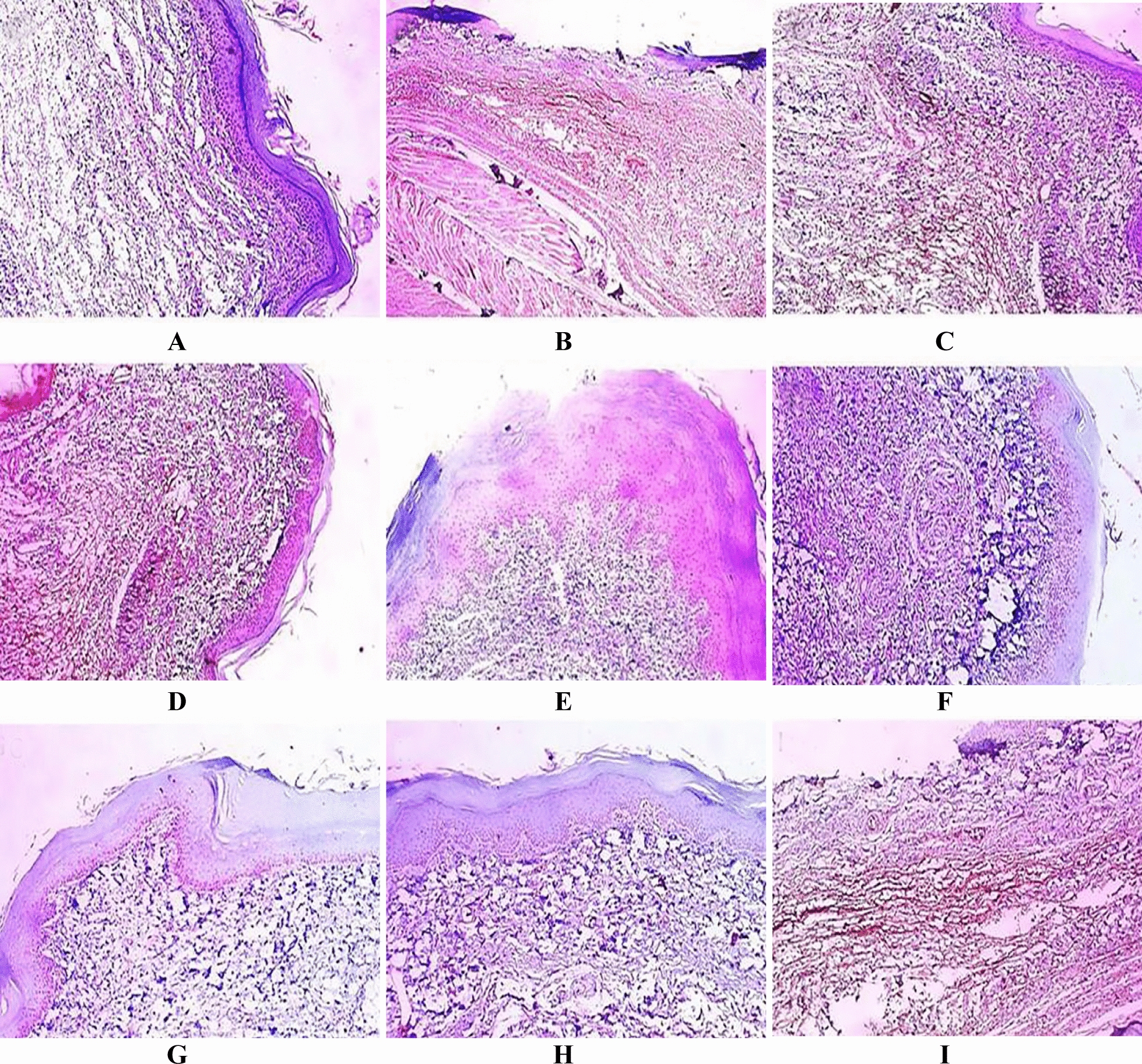
IL-1β immuno-stained paw skin sections [×100]. **A** Group I (negative control) showed negative IL-1β expression score 0. **B** Group II (positive control) showed marked IL-1β expression with score 4. **C** Group III (rats pre-treated with free extract 25 mg) showed moderate IL-1β expression with score 2. **D** Group IV (rats pre-treated with free extract 50 mg) showed moderate COX-2 expression with score 1. **E** Group V (rats pre-treated with Se NPs loaded with extract 25 mg) showed weak IL-1β expression with score 1. **F** Group VI (rats pre-treated with Se NPs loaded with extract 50 mg) showed negative expression with score 0. **G** Group VII Group (rats received free extract 50 mg only) showed negative IL-1β expression with score 0. **H** Group VIII (rats received placebo SeNPs only) showed negative IL-1β expression with score 0. **I** Group IX (rats pre-treated with placebo SeNPs) showed strong IL-1β expression with score 3

Figures 11 and 12 demonstrate the immunostaining for COX-2 and IL-1β in the paw skin tissues of the various experimental groups.

## Discussion

Regarding the ultraviolet–visible spectroscopy (UV–Vis) analysis, our findings are ultimately similar to those reported by Touliabah et al., who indicated the formation of SeNPs using the aqueous extract of *Polycladia myrica* showed a ruby red color [12]. Figure 2 showed a gradually increasing increment of absorbance from 350 to 400, and after 48 h, the absence of a striking colour deepening that would indicate reaction saturation indicates the particles may be evenly scattered throughout the reaction medium with just minimal aggregation [40]. The color change from light brown to dark brown was caused by treating *P. crinita* aqueous extract with Na_2_SeO_4_ served as the initial indicator for PCSeNPs. This alteration, which was detected by UV–visible spectroscopy, is primarily dependent on the frequency and width of the surface plasma on absorption, the dimension and shape of the metal nanoparticles, as well as the dielectric constant of the metal and its surrounding medium [41, 42]. The metals in the tested solution that support the passage of electrons from a low to a higher energy state react with UV radiation in this reaction, and the SPR is produced [43].

By employing FTIR analysis, the bioactive substances in the brown algal aqueous extract of *P. crinita* have been identified along with their contribution to SeNPs’ reduction, capping, and stabilization. The existence of C=O from polysaccharide moieties is confirmed by FTIR of the algal aqueous extract at 1636 cm^−1^, whereas the broadband at 2104.3 cm^−1^ is connected to the –NCS stretching of fucoidan from biomass and explains the sulfone stretching peak [44]. The OH and NH stretching vibrations were related to the broadness of bands at 3435.2. The bending C=C of an alkene is what induced the steep absorption peak at 876.4 cm^−1^. While the peak at 1423 cm^−1^ is correlated with stretching CO of carboxylic salts, the peak at 1094 cm^−1^ related to stretching C–O or bending C–H or stretching CN of primary amines [45], while the peak at 1198 cm^−1^ correlated with stretching C–O of the alcohol and overlapped with stretching CN of tertiary amines [46]. Moreover, the broad peak at 2104 cm^−1^ denotes C–H bending of aromatic compounds [44], while the absorption peak at 1636 cm^−1^ is associated with water adsorption in the sample [47]. The S–H group of thiol-containing compounds was believed to have been stretched as a result of the weak absorption peak at 2527 cm^−1^ [46]. Finally, the N–H and O–H groups of various amino acids observed in the aqueous extract of brown algae corresponded to the broad and strong peak that occurred at 3435 cm^−1^ [29, 47, 48]. The most frequent functional groups identified in the algal aqueous extract that are responsible for producing NPs, according to Jena et al., are the –NH2–, –C=O–, and –SH– groups [48]. The role of bioactive substances identified in the algal aqueous extract, comprising primary and tertiary amines, polysaccharides, amino acids, and others, has been confirmed by the FTIR analysis to function as reducing, capping, and stabilizing SeNPs. By comparing the FTIR (Figs. 1 and 3), the high degree of similarity proves the capping of the algal extract component for PCSeNPs [12], and the shifting in some peaks, like the shifted beak 1418 that indicated the adsorption of CO_2_ and CO_3_^−2^ on the PCSeNPs surface, illustrates the interaction between the algal extract component and the formed SeNPs [49]. Additionally, the development of absorption peaks in the 467–693 cm^−1^ regions indicates that Se–O has been successfully synthesized [12]. The vibration of C–H bending is associated with the absorption peak at 1323 cm^−1^ [50].

Shape, size, and distribution are three characteristics that are frequently associated with NP activity [51]. As a result, it is critical to identify NPs’ size and shape. The bioactive substances detected in the aqueous extract of the macroalgae *P. crinita* have the ability to create smooth spherical and semispherical forms, as indicated by the TEM study in Fig. 4. SeNPs with a size range of 5.55–29.48 nm were obtained for PCSeNPs in the current study. This obtained size range of PCSeNPs is anticipated to have significant activity for a variety of applications. Our results are in harmony with those of other researchers who synthesized SeNPs from *Ulva lactuca* and *Polycladia myrica*, respectively, and found that the formed SeNPs were spherical and semispherical smooth particles with small sizes that achieved the best results in the applied applications [12, 52].

The basic composition of the biosynthesized PCSeNPs is identified using the EDX analysis. The EDX analysis demonstrated the excellent purity of the phyco-synthesized SeNPs, which comprise Se and O ions, indicating the successful synthesis of SeO by utilizing metabolites detected in the aqueous extract of the brown alga *P. crinita*. Additionally, the peak of Se and O at bending energies between 0.5 and 1.5 keV provides further evidence that SeO has been effectively synthesized [12]. According to the quantitative examination, the sample had weight percentages of Se and O ions of 1.09 ± 0.13 and 36.62 ± 0.60%, respectively, and atomic percentages of 0.20 ± 0.02% and 32.42 ± 0.53%, respectively. The EDX profile of SeNPs created using aqueous extracts from *Polycladia myrica* appears comparable [12]. The presence of C shows that algal metabolites have been attached to the surface of SeO-NPs. In line with the present investigation, it was suggested that the biomolecules bound to the surface of SeO by the brown alga *Polycladia myrica* were the source of the carbon found in the EDX chart of SeONPs [12].

When compared to JCPDS card number 65–1290, the acquired peaks from the PCSeNPs XRD crystallinity illustration demonstrated that the phyco-synthesized SeO-NPs had a crystalline structure [12]. Debye Scherrer’s equation can be employed to determine the size of SeO-NPs produced during biosynthesis based on the width of the sharp XRD peak, which has a 2θ value of 32.13°. Based on XRD analysis, the data revealed that the mean crystal size of photosynthesized SeO-NPs was 29.05 nm. For greenly synthesized SeNPs, several naturally occurring organisms reported comparable XRD spectra [12].

The SEM micrographs of the synthesized nanoparticles revealed that they were polydispersed, spherical in shape, highly distributed, and had an average size of 21.05–22.95 nm, which is consistent with previous research [12]. The crowded background in SEM images indicated the presence and capping of PCSeNPs with the algal extract component, which was confirmed with the FTIR curves. Zeta (ζ), rather than the actual charge on each molecule, denotes an approximation of the electric double layer that was formed in the media by the adjacent ions. Because of the strong electrostatic attraction between the particles, NPs with values between + 30 mV and − 30 mV frequently demonstrate stability. The PCSeNPs that were synthesized had a verified negative charge, demonstrating improved stability of the NPs without accumulation. In the current investigation, zeta potential was used to determine the potential of the nanoparticles. The zeta potential was induced at a voltage of − 13.9 mV.

Marine algae have long been recognized as precious resources for molecules with strong functional bioactivities. Additionally, it has been demonstrated that brown algae are capable of producing a wide range of secondary metabolites with a wide range of structural patterns and functions [53]. Numerous studies have been conducted on the secondary metabolites of the genus *Cystoseira*. In addition to being thoroughly studied, polysaccharides [54], terpenes [55], lipids and sterols [56], and numerous diterpenoids have all been isolated as linear diterpenes or acyclic and cyclic meroditerpenoids [57]. These compounds may contribute to the reported pharmacological activity and are proposed to function cooperatively to exert it. According to reports, these kinds of chemicals have a range of significant pharmacological properties, such as anti-inflammatory properties [58]. In this study, we have demonstrated that *Polycladia crinita*, formerly referred to as *Cystoseira crinita*, has strong inhibitory efficacy against the synthesis of pro-inflammatory mediators such as PGE2, COX-2, and IL-1 in carrageenan-induced paw edema. Additionally, pretreatment groups with 25, 50 mg/kg of *P. crinita* extract showed a substantial decrease in the MDA levels (P < 0.00, 26.9%, and 51.68% decrease, respectively), indicating that it has a potent antioxidant effect. Moreover, the groups pretreated with SeNPs loaded with the *P. crinita* extract significantly suppressed the MDA levels (P < 0.00, 54.77%, and 65.08% decreases, respectively), relative to the positive control group. Our results agree with previously published research that investigated the anti-inflammatory and antioxidant features of *Cystoseira* spp. [59–63].

Previous studies on the anti-inflammatory activities of *Cystoseira* algae extracts have primarily examined the carrageenan-induced rat paw edema model [36, 64–66]. According to Mhadhebi et al., the edema caused by carrageenan develops in two phases. The first phase (which lasts 90 to 180 min) of the inflammation is brought on by the secretion of histamine, serotonin, and other chemicals. Prostaglandins, proteases, and lysosomes are released, as well as kinin-like compounds, during the later phase (about 270 to 360 min). Furthermore, organic extracts of *Cystoseira crinita* decreased hindpaw edema and demonstrated a dose-dependent anti-inflammatory effect. However, the results varied depending on the extract's early/later phases [65].

According to previous studies, the anti-inflammatory properties of genus *Cystoseira* are mostly attributed to polyphenols and sulfated polysaccharides such as fucoidan [54, 66–68]. Apostolova et al*.* reported that fucoidan has been shown to have anti-inflammatory effects by scavenging free radicals and inhibiting the release of TNF-α, IL-1β, IL-6, prostaglandin E2 (PGE2), and nitric oxide (NO) [69]. The release of pro-inflammatory cytokines like IL-6 and the manifestation of COX-2 are both markedly decreased, while the level of the anti-inflammatory cytokine IL-4 is simultaneously increased in the carrageenan-injected rat paw tissues, suggesting that this pharmacological effect may be related to the seaweed composition [70].

In this research, we studied the possible anti-inflammatory and antioxidant properties of phyco-synthesized selenium nanoparticles. Our results demonstrated that the synthesized NPs had greater activity than the extract from *Polycladia crinita*. Since it is impossible to transfer bioactive components to the intended location, one goal of employing NPs in nanomedicine is to transport chemicals, namely bioactive molecules, to the targeted cells and tissues. The integration of macroalgae with NPs may increase their therapeutic effectiveness and decrease the ultimate cytotoxicity of the transported materials even further [71]. They are advantageous in that they lower medication concentrations, decrease toxic effects, increase solubility, protect pharmaceuticals during circulation, and prevent drug decomposition [72]. Remarkably, algae extracts are frequently used as stabilizers and catalysts for the manufacture of metallic NPs, as well as chemicals that are encapsulated in NPs for the treatment of various medical conditions [73, 74]. Another hot topic right now is the green synthesis of NPs, which enables the full utilization of their medicinal capabilities while avoiding the usage of highly hazardous ingredients and demonstrating to be an economical and beneficial method [75].

The present study had some limitations. First, only male rats were involved in the experimental model for inflammation induction. Most studies performed on novel biological active compounds aim to test the activity of such compounds, regardless of the sex factor. Future studies are recommended to evaluate the effect of hormonal factors as well as other factors, including diet and the time of the sample collection, on the activity of the tested biomolecules. Second, the exact mechanisms by which the extract of *P. crinita* exerted its relevant anti-inflammatory effect were not clearly identified. Further experimental assays could be conducted to determine the levels of other inflammatory markers. Although this current study had some limitations, there were also strengths. This is a pioneering study evaluating the anti-inflammatory activity of *P. crinita* extract using different concentrations. Also, we studied the anti-inflammatory activity of biosynthesized *P. crinita* selenium nanoparticles (PCSeNPs) as a unique drug delivery strategy. The choice of selenium nanoparticles (SeNPs) was exceptional owing to their characteristic biological activity, chemical stability, and low toxicity.

## Conclusions

The present study is the first to describe the role of *Polycladia crinita* extract and the biosynthesized *P. crinita* selenium nanoparticles (PCSeNPs) as antioxidants and inhibitors of the release of pro-inflammatory cytokines. These results demonstrate that the brown seaweed *P. crinita* extract and PCSeNPs have powerful antioxidant and anti-inflammatory properties. These unique characters that are combined in a single alga suggest that this group of marine algae is interesting for research in the field of inflammation management.

### Supplementary Information

**Additional file 1: Figure S1.** Experimental design and animal groups.

## Data Availability

All data generated or analyzed during this study are included in this published article.
