# Supplementary figures and images for: New investigation of anti-inflammatory activity of Polycladia crinita and biosynthesized selenium nanoparticles: isolation and characterization
Source: Microb Cell Fact. 2023 Sep 5;22:173. doi: 10.1186/s12934-023-02168-1 (PMC10478239; doi:10.1186/s12934-023-02168-1)

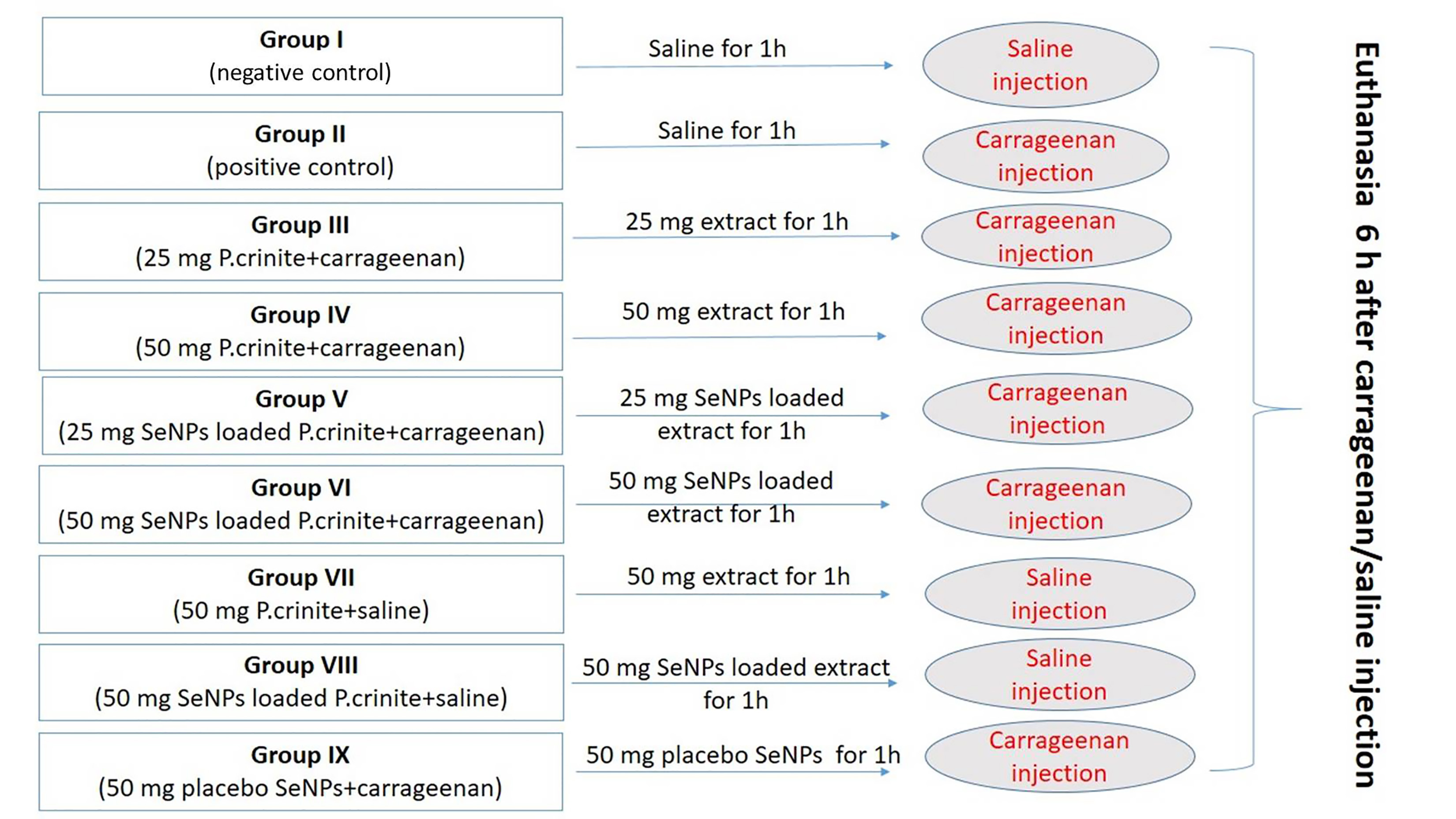

Supplement: Supplementary file 1 — Additional file 1: Figure S1. Experimental design and animal groups. [file 12934_2023_2168_MOESM1_ESM.jpg]
